# Cryo-preserved porcine kidneys are feasible for teaching and training renal biopsy: “the bento kidney”

**DOI:** 10.1186/2047-1440-1-5

**Published:** 2012-05-02

**Authors:** Kenjiro Konno, Koichi Nakanishi, Shuji Hishikawa, Hozumi Tanaka, Norishige Yoshikawa, Yoshikazu Yasuda, Eiji Kobayashi, Alan Lefor

**Affiliations:** 1 Center for Experimental Medicine, Jichi Medical University, Tochigi, Japan; 2 Department of Surgery, Jichi Medical University, Tochigi, Japan; 3 Medical Simulation Center, Jichi Medical University, Tochigi, Japan; 4 Department of Pediatrics, Wakayama Medical University, Wakayama, Japan

## Abstract

**Background:**

The use of patients as the primary teaching modality for learning procedures is being questioned. While there have been advancements in the technology used for performing needle biopsies in both native and transplanted kidneys, there has been little advancement in teaching and training tools. We have developed a portable *ex-vivo* kidney, the Bento Kidney, using cryo-preserved porcine kidneys for teaching this procedure.

**Methods:**

The kidney is thawed, perfused by a pump, covered with skin for realistic haptic feedback, and then used with existing biopsy technology to teach the technique.

**Results:**

Thirty porcine kidneys were used in this pilot research, and nine were shipped to physicians at a distant facility. Renal biopsy was then performed using a core biopsy needle and ultrasound guidance. There was some leakage of fluid from all kidneys noted. All trainees felt that the model was realistic, and judged at a mean score of 8.7 (SD 0.8) on a scale of 1 (not useful) to 10 (very useful).

**Conclusions:**

This feasibility study demonstrates that cryo-preserved porcine kidneys can be successfully used to teach and train renal biopsy techniques, and provides haptic feedback as well as realistic real-time ultrasound images. Further large scale studies are needed to demonstrate value from the educational point of view for nephrology and transplantation.

## Background

It is well regarded that percutaneous renal biopsy (PRB) is an integral part of the clinical practice of nephrology [1]. There may be less time to learn allograft biopsies due to less frequent hemorrhagic complications than native kidney biopsies [2]. However, there is a reported 10% complication rate for outpatient needle biopsy of transplanted kidneys, resulting in re-hospitalization [3]. The introduction of automated biopsy needles and ultrasound guidance has improved this procedure. The importance of adequately teaching PRB has been underscored by the shift of this procedure in some institutions from nephrologists to radiologists [1,4]. While new technology has simplified the conduct of PRB and eliminated the need for open surgical biopsy, there remains a need for adequate teaching models other than live animals and patients [4].

In order to educate the future physicians and transplant surgeons, new approaches to education are being used, especially teaching in non-clinical environments [5]. Patients and medical professionals have expressed great concern about using patients for the primary teaching of procedures, and technology has provided a range of alternatives. In Japan it is legally prohibited for a medical student to perform any invasive procedure on a patient other than simple venipuncture. Thus, teaching procedural skills to both students and newly trained doctors in Japan has been impossible until the recent introduction of various simulators.

We have developed an *ex-vivo* kidney model, using cryopreserved porcine kidneys, which allow teaching the technique of PRB providing realistic haptic feedback. The model is simple to use, and is easily transported. The entire model is contained in a single box, reminiscent of the traditional Japanese meal-in-a-box, and is thus referred to as “The Bento Kidney”. This study was undertaken to evaluate the feasibility of using the Bento Kidney for teaching the technique of PRB.

## Methods

Fifteen experimental miniature pigs were used for a variety of experiments at Jichi Medical University. All experiments were approved by the Institutional Animal Care and Use Committee. The pigs weighed 24-45 kg each. At the completion of planned experiments, just prior to euthanasia, Heparin (10000U IV) was administered. After euthanasia, the kidneys were harvested with a 3 cm length of renal artery, renal vein and ureter. The kidneys were preserved at -20°C until shipment or use.

After freezing, the kidneys were placed individually in plastic boxes. A total of nine kidneys were shipped, in the frozen state, to Wakayama Japan, located about 600 km away, at a cost of about US$9 for each kidney. The remaining kidneys were used at Jichi Medical University.

**Figure 1:**
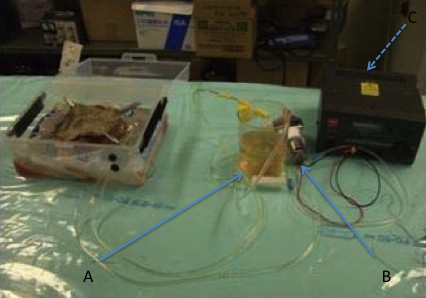
**The cryopreserved kidney is perfused by a peristaltic pump after thawing, and contained entirely in a “Bento” lunchbox.** (A = Fluid reservoir, B = Peristaltic pump, C = Power Supply).

**Figure 2:**
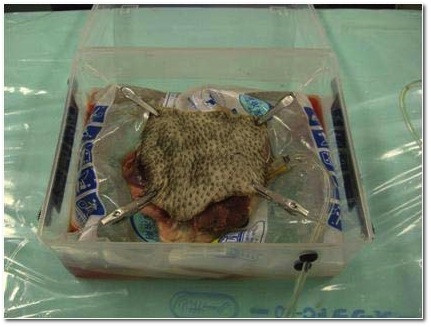
The kidney is covered by porcine skin, to provide haptic feedback and a more realistic training model.

The kidneys were stored a variable amount of time (3 days to 7 months) before use. Before use, a kidney was placed in lukewarm water, thawed at room temperature, and connected to a pulsatile pump (50 mL/min) using plastic tubing to connect to the artery and vein remnants (Figure 1). After assuring perfusion, and noting the egress of fluid from the ureteral stump, the kidney was covered with harvested porcine skin, to improve the reality of the haptic feedback for the trainee (Figure 2).

**Figure 3:**
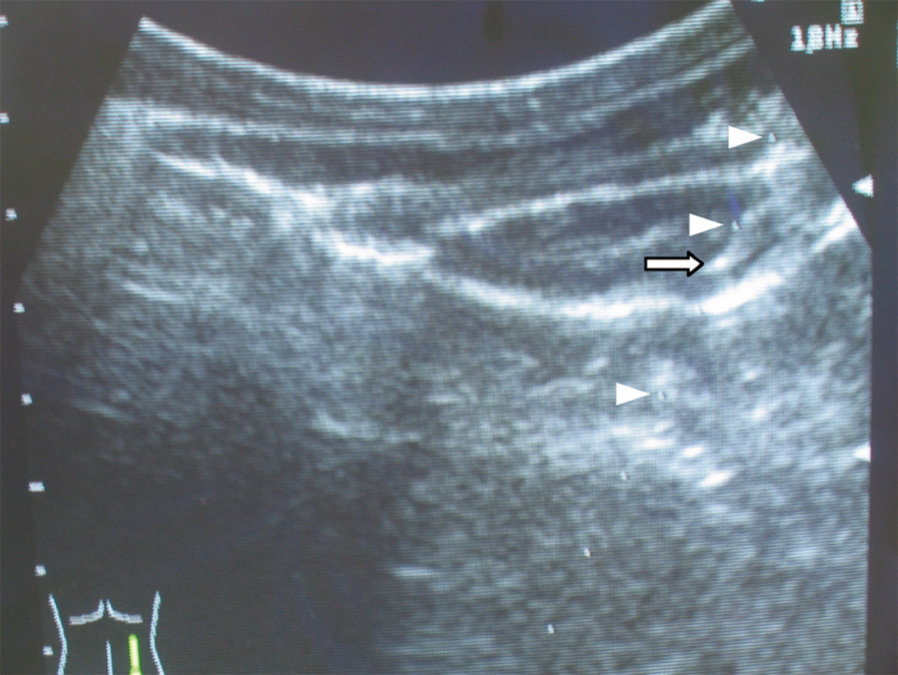
**Ultrasound image of the perfused Bento kidney.** Arrowheads show the progress of the biopsy needle, with the tip indicated by the arrow.

Renal biopsy was then performed using a standard (14 G) biopsy needle with ultrasound guidance in the usual manner, with each trainee receiving one-on-one supervision from an experienced clinician. A typical ultrasound image is shown in Figure 3.

## Results

All of the kidneys were used to teach the technique of PRB. The kidneys were used in a total of five separate training sessions, and a total of 32 trainees participated in the simulation program. Survey data was obtained from each participant. In three instances, teaching was done without using a perfusion pump. The nine kidneys that were shipped frozen all functioned well in this model.

**Figure 4:**
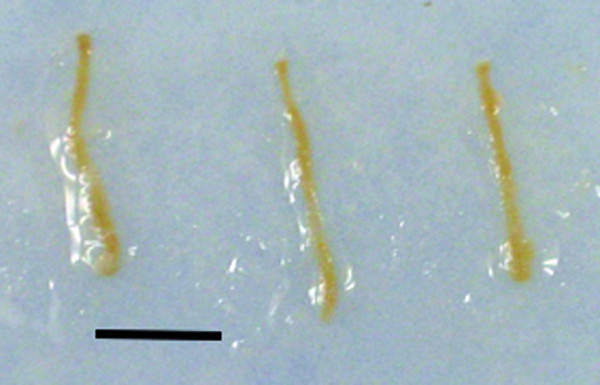
**Biopsy specimens obtained from a session with the Bento kidney.** The black bar is 1 cm in length.

PRB was subjectively considered well simulated in this system. The view of the kidney using the ultrasound guide was excellent (Figure 3) and needle visibility was also very good. There was excellent haptic feedback during the procedure. It subjectively resembled that of the human very well. Kidney specimens were easily obtained (Figure 4). In this system, not only the procedure for obtaining the biopsy specimens, but also processing the samples for histological examination could be practiced.

Following performance of the biopsy, the perfusion fluid leaked in all of the kidneys, ranging from a small amount to a large amount. If the puncture site traversed a major vessel in a kidney connected to the perfusion pump, then leakage occurred due to an obvious lack of clotting.

The model functioned well in all instances, providing a useful ultrasound image. In a questionnaire given to the trainees, the usefulness of the system was judged on a scale from 1 to 10 with 10 being most useful. The mean score was 8.7 (SD 0.8). All respondents felt that the system provided an excellent educational experience, regardless of their background or previous biopsy experience.

## Discussion

The Japanese word “*Bento*” is sometimes translated to mean a lunchbox, but the implications of this word are much deeper than a simple lunchbox. *Bento* actually means a complete self-contained meal that contains food cooked in specific ways and of certain colors, then neatly packaged for easy transport. In a similar manner, the Bento kidney is a complete teaching tool in a box that is easily transported just like its namesake, the *Bento* “lunchbox”.

In view of concerns about using patients as a primary teaching tool, future clinical teaching must include other environments such as using mechanical simulators (the “dry” lab), and animal procedures (the “wet” lab). There has been considerable academic interest in the use of simulation for teaching both cognitive skills such as care of the patient in cardiac arrest, as well as procedural skills such as laparoscopic surgery.

Simulation may be a way to reduce dependence on animals for the teaching of some procedures. A fundamental principle in the conduct of animal experimentation is referred to as the “3-Rs” being Refinement, Reduction and Replacement. To comply with these principles, we are exploring ways to reduce the dependence on live animals for the teaching of procedures. One approach is to use many different parts of an animal that might otherwise be simply wasted at the end of a single procedure.

There has been some advancement in PRB technology, resulting in the near elimination of open surgical biopsy, largely through the widespread use of ultrasound guidance and biopsy needles [1,4]. One study has shown that multiple passes with the biopsy needle (more than two) does not increase the complication rate compared to one or two passes [6]. This result is important for teaching this procedure. A more recent advance has been the use of automated spring-loaded biopsy needles, further improving the safety and ease with which the procedure is performed [7].

However, there has been little evolution in technology used for teaching PRB. The use of automated biopsy needles and ultrasound imaging has revolutionized the conduct of the procedure, but does not represent a change in teaching the procedure. The Bento Kidney represents a new way to teach this important technique that can be used easily with available technologies.

This study shows that this model is feasible, and portable since the kidneys are easily shipped. The use of the pulsatile perfusion pump is not mandatory for the haptic feedback resulting from puncturing the renal parenchyma. By using the perfusion pump, not only is the vasculature realistically seen on ultrasound, but vascular puncture results in leakage of fluid, somewhat analogous to the *in vivo* situation, enhancing the feedback from this model.

The current study establishes that this model is feasible and easily transported to other institutions. Future studies using this model will be initiated to determine the educational effectiveness of the model and to compare it further with actual clinical practice. Future studies are needed to determine the amount of training to achieve clinical competence, and to evaluate the effectiveness of the model for teaching biopsy techniques (e.g. cortex vs. medulla) and localization techniques (e.g. upper vs. lower pole).

The simulation offered using this model is not a perfect replica of renal biopsy on a patient. This model has no motion of the practice kidney caused by breathing, which makes renal biopsy on a patient difficult. The skin of the pigs was firmer than that of a human. Therefore, it was necessary to incise the skin more than in the clinical setting. Subcutaneous tissue was not around the kidney, and kidney moved easily in some cases.

It is acknowledged that there may be alternative ways to teach this technique. A mechanical model might be useful, but would be limited by the lack of authentic haptic feedback. A live animal model may be useful in some circumstances, but would likely be prohibitively expensive and utilize many animals. The model described here provides a realistic experience, at a reasonable cost, and respectful of the 3R principle of animal ethics.

In renal allograft biopsies, hemorrhagic complications have been known to be less frequent than in native kidney biopsies, partly due to efficient external compression to prevent or stop bleeding from the puncture site [1]. However, while gross hematuria is the most frequent problem, this does not compromise the patient or graft survival showing it to be a safe outpatient procedure [2]. Having demonstrated that this training model is easy to use providing haptic feedback as well as realistic ultrasound images, further studies are underway to determine its educational value.

## Conclusion

This study has shown that cryopreserved porcine kidneys are feasible as a model for teaching the technique of percutaneous kidney biopsy. These kidneys can be harvested from animals used for other experiments and thus respecting the fundamental principles of Reduction, Refinement, and Replacement in animal research.

## Competing interests

All authors declare that they have no competing interests.

## Authors’ contributions

KK: Study conception, study design, training sessions, data analysis, manuscript preparation, approval of final manuscript. KN: Study design, training sessions, data collection, approval of final manuscript. SH: Study design, training sessions, data collection, approval of final manuscript. HT: Study design, training sessions, data collection, approval of final manuscript. TY: Study design, training sessions, data collection, approval of final manuscript. YY: Study conception, Study design, approval of final manuscript. EK: Study conception, study design, approval of final manuscript. AL: Study conception, study design, data analysis, preparation and approval of manuscript.
